# Secondary caries and marginal adaptation of ion-releasing versus resin composite restorations: a systematic review and meta-analysis of randomized clinical trials

**DOI:** 10.1038/s41598-022-19622-6

**Published:** 2022-11-10

**Authors:** Eman H. Albelasy, Hamdi H. Hamama, Hooi Pin Chew, Marmar Montaser, Salah H. Mahmoud

**Affiliations:** 1 Conservative Dentistry Department, Faculty of Dentistry, Mansoura University, Algomhoria Street, Mansoura, Aldakhlia 35516 Egypt; 2 Research Visiting Scholar, Minnesota Dental Research Centre for Biomaterials and Biomechanics, School of Dentistry, University of Minnesota, Minneapolis, MN 55455 USA; 3 Restorative Dentistry Department, Faculty of Dentistry, New-Mansoura University, New-Mansoura, Egypt; 4 Minnesota Dental Research Centre for Biomaterials and Biomechanics, School of Dentistry, University of Minnesota, Minneapolis, MN 55455 USA; 5 Conservative Dentistry Department, Faculty of Dentistry, Horus University, New-Damietta, Egypt

**Keywords:** Health care, Medical research

## Abstract

This systematic review was aimed to evaluate occurrence of secondary caries and marginal adaptation in ion-releasing materials versus resin composite. Electronic search of PubMed, Scopus, and Open Grey databases with no date or language restrictions until May 21st, 2021, was conducted. Randomized clinical trials that compared ion-releasing restorations versus resin composite were included. For quantitative analysis, a random-effects meta-analysis with risk difference as an effect measure and a 95% confidence interval was used. Quality of evidence was assessed using The Grading of Recommendations, Assessment, Development, and Evaluation criteria. The risk of bias was evaluated using the Cochran Collaboration Risk of Bias tool. The inclusion criteria were met by 22 studies, and 10 studies were included in the meta-analysis. Three follow-up periods (1 year, 18 months–2 years, and 3 years) were evaluated. The overall quality of evidence for secondary caries and marginal adaptation outcomes was low. The results of the meta-analysis showed no significant difference (*p* > 0.05) in both outcomes between ion-releasing materials and resin composite. The occurrence of secondary caries was not dependent on the nature of the restorative material. It is more likely a complex process that involves the same risk factors as primary carious lesions.

## Introduction

Over the last decade, remarkable advances in resin composite formulations have been made to address clinical challenges. Bulk-placement techniques, new filler formulations, and simplified adhesion protocols have resulted in a more user-friendly application^1,2^. However, the clinical problems of technique sensitivity, polymerization shrinkage, and lack of antibacterial properties remained unchanged^3–5^ and similarly, the main reasons for its failure remain to be secondary caries and bulk fractures^1,6^.

Secondary caries can be defined as caries lesions at the margins of existing restorations^7^ or caries associated with restorations or sealants (CARS) (secondary caries and caries around restorations are used synonymously in this review)^8,9^. The complexity of caries around restorations is related to its multifactorial origin, combining the pathological pathway of primary carious lesions with the influence of the formulations of different restorative materials^9^. It has been reported that thicker biofilms accumulate around resin composite than glass ionomer restorations^10^. In vivo plaque studies have also shown that the levels of lactic acid-producing bacteria are significantly higher around resin composite restorations than on either amalgam or glass ionomer restorations^11,12^. Therefore, fluoride-releasing materials that possess remineralization and/or antibacterial properties have gained popularity in recent years^13^ with the hope of preventing secondary caries formation.

Conventional glass ionomer cement (GICs) and its evolutions such as: high-viscosity glass ionomer (HV-GIC), resin-modified glass ionomer (RMGIC), and compomers are the most frequently used fluoride-releasing restorative materials. An inherent disadvantage of GIC is its low fracture toughness, which limits its clinical applications to low load-bearing areas such as the buccal and lingual surfaces. Nevertheless, increasing the powder-liquid ratio, and modifications in its chemical composition have shown to lead to improved physical properties and prolonged clinical survival^14,15^.

Modified versions of the conventionally set GIC such as HV-GIC were introduced with the hope of extending the indications of GIC to include load-bearing areas on posterior teeth to provide an alternative for patients with limited resources^16–18^. Promising 10-years clinical results have recently emerged for HV-GIC used in class I and II restorations, where no restoration had to be replaced due to unacceptable clinical wear^19^. In addition to HV-GIC, glass hybrid materials such as Equia Forte were introduced in 2015. According to the manufacturer, these materials are modified with highly reactive glass particles of different sizes to significantly increase their mechanical properties^20,21^.

Nonetheless, the clinical indications of GIC and its evolutions in multiple-surface restorations in the stress-bearing posterior regions of the mouth are still limited due to their poor fracture toughness, tensile strength, wear resistance, and hardness. A recent systematic review reported that the annual failure rates of approximal or multi-surface GIC restorations were greater than those of single-surface occlusal restorations^22^. A solution to counteract this limitation of GIC is to incorporate resin composite restorations (which have superior mechanical properties than GIC) with reactive fillers that can protect the tooth against secondary caries^23^. Up to press date, there are several new commercially available ion-releasing composites with claimed bioactivity such as ACTIVA™ BioACTIVE-RESTORATIVE™ (Pulpdent Corporation, Watertown, MA, USA), Cention N (Ivoclar Vivadent, Schaan, Liechtenstein), and Surefil one (Dentsply Sirona). These materials are relatively recent additions to the realm of ion-releasing materials, that are claimed by their respective manufacturer, to release sufficient amounts of ions other than fluoride to promote remineralization^24–26^ around restorations. Tiskaya et al. ^27^, reported significant release of Al^3+^ and Ca^2+^ ions from Cention N and Activa Bioactive in acidic media of pH 4, which in turn indicate an ability to protect against secondary caries.

Clinical investigations regarding their ability to inhibit caries around restorations are scarce in the current literature. While in vitro studies have shown that fluoride-releasing restorative materials such as GICs can inhibit tooth demineralization adjacent to restoration margins^28–30^, the caries inhibitory effect of these new ion-releasing materials remains unclear. Therefore, this systematic review and meta-analysis were aimed to answer the following question: Is there a difference in the occurrence of secondary caries and marginal adaptation in ion-releasing restorations compared to resin composite?

## Materials and methods

The recommendation of the preferred reporting items for systematic reviews and meta-analysis (PRISMA) were followed in this review^31,32^.

### Eligibility criteria and PICO question

The research question was as follows: Is there a difference in the incidence secondary caries and marginal adaptation in ion-releasing restorations compared to resin composite?

The following PICO questions were established:

- Population: patients with permanent dentition in need of restorations.
- Intervention: ion-releasing restorations. From here forth, the term ‘ion-releasing’ will be used in this article to encompass fluoride and all other ion-releasing materials. All GIC derivatives including (RMGIC, HV-GIC, conventional GIC, and glass hybrid), polyacid-modified composite (compomer), giomer, and any material stated by the manufacturer to be capable of ion-release will be in the intervention group.
- Comparison: the intervention should be compared with a resin composite restoration applied in conjunction with any adhesive system.
- Outcomes: caries around restorations and marginal adaptation.

### Inclusion criteria

1. Randomized clinical trials in patients with permanent dentition comparing an ion-releasing material to resin composite in any form of cavities (Black’s Class I, II, V) and non-carious cervical lesions (NCCLs).
2. Parallel or split-mouth studies.
3. A minimum follow-up period of 1 year.
4. Evaluation criteria: FDI criteria and/or USPHS.
5. The investigated materials must be commercially available. Any study investigating discontinued products was excluded.

### Exclusion criteria

1. Editorial letters, pilot studies, historical reviews, literature reviews, systematic reviews, in vitro studies, cohort, observational and descriptive studies, such as case reports and case series.
2. Randomized clinical trials were excluded if.
  - Ion-releasing materials were compared to each other with no resin composite restoration as a reference for comparison.
  - Restorations were done on primary teeth,
  - The follow-up period was less than 1 year.

### Information source and search strategy

An electronic search within the following databases (Medline via PubMed and Scopus) was conducted until May 21st, 2021. Grey literature was searched through the Open Grey database http://www.opengrey.eu/.

The following keywords were used in the electronic search: “FDI criteria AND randomized clinical trial”, “modified USPHS criteria AND randomized clinical trials”, “Secondary caries OR caries adjacent to restorations and randomized clinical trials”, “marginal adaptation and randomized clinical trial”, “ion releasing restorations OR bioactive resin composite OR bio interactive restorations AND clinical trials”. To identify ongoing clinical trials, we also searched the ClinicalTrials.gov website. The outcome of the search among the abovementioned databases was comprehensively checked and duplicated results was excluded.

To minimize publication bias, no language or publication date restrictions were applied. Two reviewers (E.H. and H.H.) independently extracted data and assessed their eligibility and risk of bias. Any disagreements were resolved by consulting a third reviewer (H.C.).

### Study selection and assessment of eligibility

According to the search strategy, assessment of the eligibility of trials was performed by the two reviewers according to the relevance of the title. Abstracts of studies that could not be excluded based on the title were retrieved and evaluated. At the final stage of evaluation, full texts were assessed to determine if they met the predetermined inclusion criteria. The included studies received an identification code composed of the first author’s last name and the year of publication.

Two reviewers extracted data from included studies such as the number of patients and restorations per group, intervention, and comparator, follow-up period, study design, evaluation criteria, adhesive strategy, cavity design, isolation technique, patient’s age, settings, and location of data collection. In studies that reported multiple follow-up periods, data from the longest follow-up were extracted. If more than one type of resin composite was used, the data were combined into a single entry. For ion-releasing restorations, GIC-based restorations (HVGIC, glass hybrid, and RMGIC) were combined into a single entry and compomer restorations were pooled together.

### Assessment of risk of bias

The Risk of Bias (RoB) of the included studies was assessed using the Cochrane Collaboration Risk of Bias Tool (version 2.0) for RCTs^33^. The six domains of the RoB Tool are assessment of random sequence generation, allocation concealment, blinding of participants and personnel, blinding of the outcome assessors, incomplete outcome data (attrition bias), selective outcome reporting, and other sources of bias. In this study, the other sources of bias domain was not included. Each entry received a judgment of low, unclear, or high risk of bias. At the study level, a study was considered at low risk of bias if all 5 domains of the RoB tool for each outcome were at low risk of bias. If one or more domains were judged to have unclear risk, the study was judged to have unclear risk. If at least one item was considered at high risk of bias, the study was considered to have a high risk of bias.

### Assessment of quality of evidence

The confidence in evidence was evaluated using the Grading of Recommendations Assessment, Development, and Evaluation (GRADE)^34^. According to GRADE, the body of evidence can be rated as high, moderate, low, or very low. The GRADE pro-Guideline Development Tool (www.gradepro.org) was used to create a summary-of-findings table.

The strength of cumulative evidence was assessed based on, the risk of bias, inconsistencies, indirectness, imprecision, and publication bias. The data were summarized in the summary of findings (Table 2). The quality of evidence for the first 4 domains may be downgraded by 1, 2, or 3 levels based on “serious or very serious risks. Publication bias may either be suspected or undetected. In the case of suspected bias, downgrading by 2 levels was made^35,36^.

### Synthesis of data

Data were analysed using Revman 5.4 (Review Manager Version 5.4, The Cochrane Collaboration, Copenhagen, Denmark). Data from included studies were either dichotomous for the *“Secondary Caries”* outcome measure or ordinal for the *“Marginal Adaptation”* outcome measure. Marginal adaptation data were dichotomized to NO representing Alpha and Bravo scores of the modified USPHS criteria, and scores 1 and 2 of the FDI criteria, or YES corresponding to Charlie and Delta scores of the modified USPHS criteria, and 3, 4, and 5 scores of the FDI criteria. Risk differences as an effect measure with 95% confidence intervals and random effects model were employed. Heterogeneity was evaluated using the Q test and I^2^ statistics, where 25%, 50%, and 75% represent low, moderate heterogeneity, and high heterogeneity respectively. For both the outcomes (secondary caries and marginal adaptation), data from 3 follow-up periods were included, i.e., 1 year, 18 months—2 years, and 3 years. For secondary caries outcome, two analyses were performed, one with all types of cavities, and one for load-bearing cavities.

## Results

### Search details

**Figure 1:**
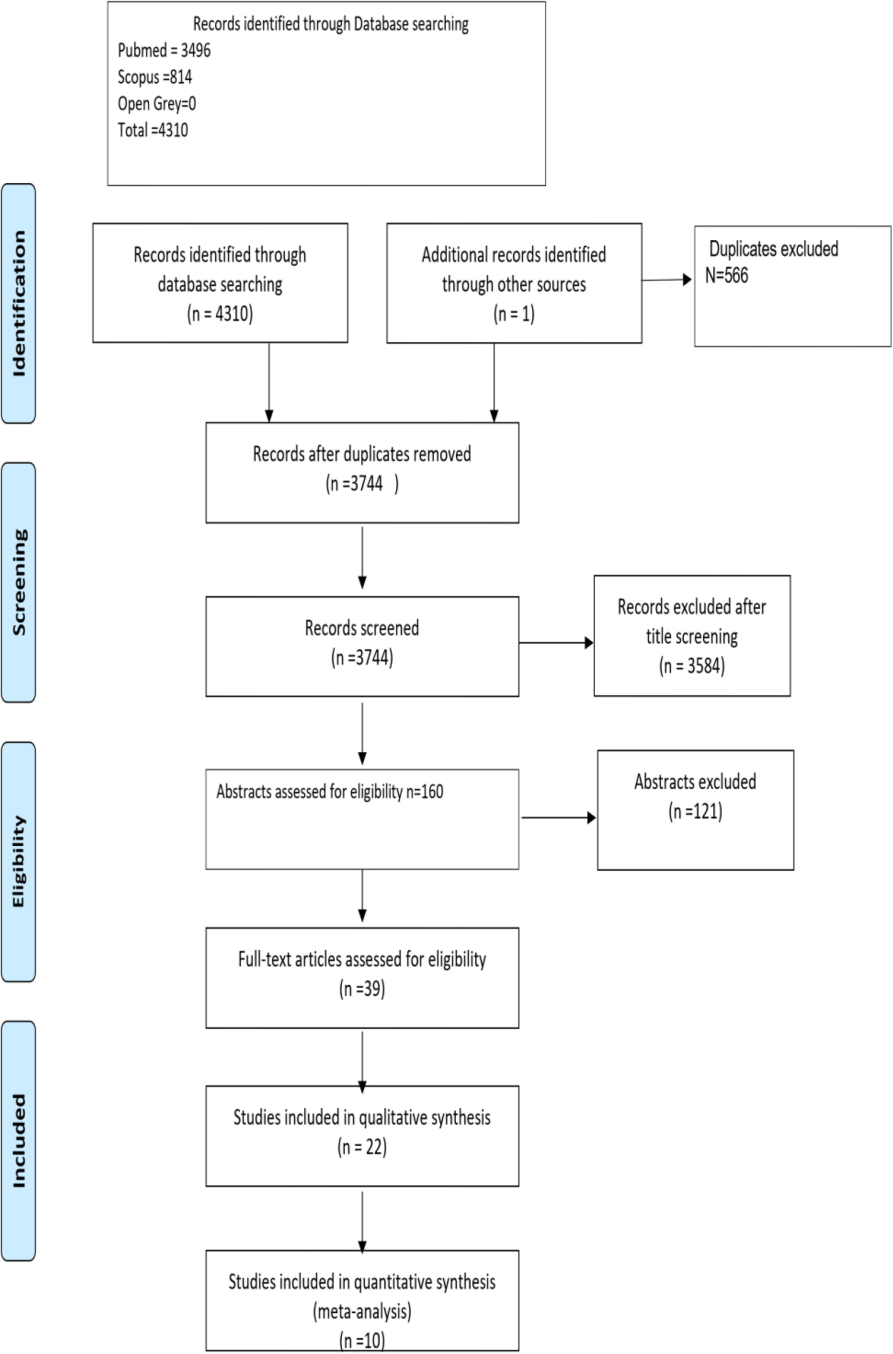
Prisma flow chart of the study selection process.

The initial search in the databases resulted in 3744 studies being identified after duplicates exclusion. After title screening, 3584 articles were excluded, and the remaining 160 abstracts were further assessed for eligibility. Articles that had multiple reports corresponding to different follow-up periods were combined into a single entry and the data of the longest follow-up were included in this study. This process culminated in 39 studies that were to be progressed to full-text analysis. Subsequent full-text analysis of these studies resulted in 22 studies that met the inclusion criteria (Fig. 1).

### Risk of bias evaluation

**Figure 2:**
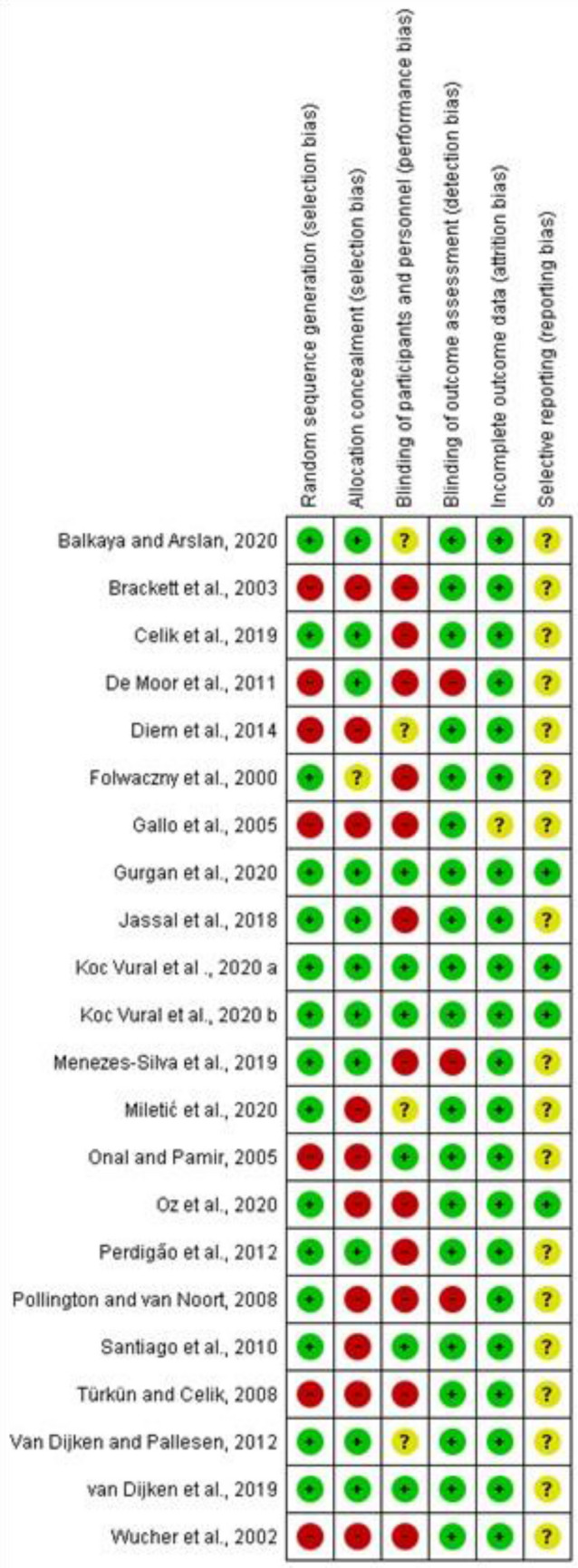
Risk of bias summary: authors' judgments about each risk of bias item for each included study. Filled Green circle Low ROB Filled Red circle High ROB Filled Yellow Circle Unclear ROB.

Overall, 3 studies were deemed to have a low risk of bias^19,37,38^, 3 studies showed^39–41^ unclear risk of bias while the remaining 16 studies had a high risk of bias. Seven studies^17,42–47^ did not report random sequence generation, while 50% of the included studies reported allocation concealment. Performance bias was unclear in the majority of studies (16 out of 22), while outcome assessment was blinded in all studies except for 3^43,48,49^. No attrition bias was noticed in any of the included studies except for one^44^, which did not adequately report the number of dropouts (Fig. 2).

### Included studies characteristics

The characteristics and methodological assessment of the 22 included studies are summarized in Table 1. In 15 of the included studies^16,19,37,38,41–44,46–48,50–53^, split-mouth design was employed while 7 studies reported a parallel study design^17,39,40,45,49,54,55^. Most of the studies employed the modified USPHS criteria for restorations evaluation except for 4 studies^16,17,50,51^ that used FDI criteria. One study^43^ used the McComb et al., criteria^56^. Five studies used HV-GIC^16,17,19,39,49^. Two studies used glass hybrid^38,51^. Resin-modified glass ionomer was used in 9 studies^37,41–43,45,50,52,54,57^, while 2 studies used conventional GIC^43,53^. Compomer (poly-acid modified composite) was used in 7 studies^40,44–48,54^. Most of the studies used nano- or micro-hybrid composite. Bulk-fill composite was used in one study^39^. Nano-filled composite was used in 2 studies^46,57^ while one study used micro-filled composite^44^. Most follow-up periods ranged between 2 and 3 years. Long-term follow-up was reported in 2 studies^19,40^ which had a follow-up period of 10 and 7 years respectively. One study^41^ was terminated after 1 year due to an unacceptable failure rate. Class II cavities were reported in 7 studies^19,39,41,47,49,51^. Class I cavities were evaluated in 3 studies ^17,19,41^. Non-carious cervical lesions were evaluated in 11 studies^16,38,42,44–46,48,50,52,53,57^. Class V carious lesions were evaluated in 4 studies^37,40,43,54^. For HV-GIC, glass hybrid, and conventional GIC, Cavity conditioner of poly-acrylic acid was used in all studies except 2 which did not report any type of pre-treatment^38,53^. For RMGIC, 2 studies used 37% phosphoric acid etching for 5 s^37,41^. Two studies used Vitremer primer^45,52^ while another study used GC cavity conditioner for RMGIC, and Ketac nano primer for nano-filled RMGIC^42,57^. For Compomer, 5 studies used self-etch adhesive (SE)^40,45,46,48,54^, while 2 studies used etch-and-rinse adhesive (ER)^44,47^.

**Table 1:** Characteristics of the included studies.

Study ID	1. Ion-releasing material	2. Type of composite	3. Evaluation criteria	4. Number of restorations/per group	5. Total number of restorations and/patients	6. Follow-up period	7. Location/settings of data collection	8. Trial design	9. Recall rate	10. Secondary caries detection
Balkaya et al.^39^	Glass hybrid: Equia Forte Fil ^a^	1.Bulk-fill resin composite: Filtek Bulk Fill Posterior ^a^ 2. Micro hybrid composite: Charisma Smart ^c^	Modified USPHS	1. Equia Forte/34 2. Filtek Bulkfill /38 3. Charisma smart /37	109/54	2 Years	Turkey/University	Parallel	100%	Visual-tactile with mirror, intraoral photographs, prob and bitewing radiographs
Gurgan et al.^19^	1. HVGIC: Equia Fil ^a^	1. Microhybrid resin composite: Gradia Direct Posterior ^a^	Modified USPHS	1. Equia Fil/40 class I, 30 class II 2. Gradia Direct Posteior/40 class I, 30 class II	140/59	10 Years	Turkey/University	Split-mouth	88.1%	Visual-tactile with mirror, coloured photographs and prob
Koc Vural et al.^37^	1. RMGIC: Riva LC ^J^	1. Microhybrid composite: Spectrum TPH3 ^e^	Modified USPHS	1. Riva LC/55 2. Spectrum TPH3/55	110/33	3 Years	Turkey/University	Split-mouth	90.91%	Visual-tactile method with mouth mirror and explorer under the dental light unit
Koc Vural et al.^38^	Glass hybrid: Equia Forte Fil ^a^	1. Nanofilled composite: Ceram X One Universal ^e^	Modified USPHS	1. Equia Forte Fil/74 2. Ceram X One/74	148/52	2 Years	Turkey/University	Split-mouth	88%	Visual with the aid of coloured photographs
Miletić et al.^51^	Glass hybrid: Equia Forte Fil ^a^	1. Nanohybrid composite/Tetric Evoceram ^c^	FDI	1. Equia Forte/179 2. Tetric Evoceram/178	358/184	2 Years	Multicenter: Croatia, Italy, Turkey, and Serbia/University	Split-mouth	90.6%	Visual-tactile with (magnification 2.5X), mirrors, and very thin (250-μm-thick) dental probes
Oz et al.^53^	Conventional GIC: Fuji Bulk ^a^	1.MFR Hybrid Composite/Gaenial Posterior ^a^	Modifies USPHS	1. Fuji Bulk/67 2. Gaenial Posterior/67	134/30	1 Year	Turkey/University	Split-mouth	93%	Visual-tactile with mirrors, probes, and air streams
Celik et al.^16^	1. HVGIC: Equia Fil ^a^	1.MFR Hybrid Composite G-aenial Posterior ^a^	FDI	1. Equia Fil /67 2. G-aenial/67	134/22	3 Years	Turkey/University	Split-mouth	82%	Visual-tactile using a mirror and an explorer
Menezes-Silva et al.^49^	1. HVGIC: Equia Fil ^a^	2. Filtek Z350 XT Universal ^b^	Modified USPHS	1. Equia Fil/77 2. Filtek Z350/77	154/154	1 year	Brazil/17 public primary schools	Parallel	94.8%	Visual-tactile with photographs, mirror, and ballpoint periodontal prob
Van Dijken et al.^41^	1. RMGIC: Activa Bioactive ^f^	1. Nanofilled composite: Ceram X ^e^	Modified USPHS	1. Activa Bioactive/82 2. Ceram X/82	164/67	1 Year	Sweden/University	Split-mouth	96.3%	Visual-tactile using mirror and explorer and radiographs one-year recall
Jassal et al.^50^	1. RMGIC: GC II LC ^a^	1. Microfine hybrid compiste/Solar X ^a^	FDI	1. GC II LC/98 2. Solar X , passive adhesive application/98 3. Solar X, rigouros adhesive application/98	294/56	18 Months	India/n.r	Split-mouth	90.81%	Visual using dental-operating microscope at 1 × magnification
Diem t al.^17^	1. HVGIC: Fuji IX GP Extra ^a^	1. Microfine hybrid Composite: Solar ^a^	FDI	1. Fuji IX GP Extra/87 2. Fuji IX GP Extra with G-coat plus/84 3. Solar /83	254/91	3 Years	Vietnam/Primary school in semi-rual area	Parallel	77.9%	Visual using headlight, natural light, and digital photographs
Van Dijken et al.^40^	1. Compomer: Dyract AP ^e^	1. Hybrid compiste/Tetric Ceram ^c^	Modified USPHS	1. Dyract AP/69 2. Tetric Ceram/70	139/60	7 Years	University	Parallel	97.1%	Visual-tactile using a mirror, and an explorer
Perdigão et al.^57^	1. RMGIC: Fuji II LC ^a^ 2. Nanofilled RMGIC: Ketac Nano ^b^	1. Nanofilled composite: Filtek Suprem Plus ^b^	Modified USPHS	1. Fuji II LC/31 2. Ketac Nano/30 3. Filtek Suprem/31	92/33	1 Year	Brazil/University	Parallel	84.8%	Visual using a mirror and intra-oral-coloured photographs at 1.5 × magnification
De Moor et al.^43^	1. Conventional GIC: Ketac Fil ^b^ 2. RMGIC: Photac Fil ^b^	1. Microhybrid compiste: Herculite XRV ^d^	McComb et al., criteria	1. Ketac Fil/35 2. Photac Fil/35 3. Herculite/35	105/35	2 Years	Belgium/Privat practice	Split-mouth	77.1%	Tactile using an explorer
Santiago et al.^52^	1. RMGIC: Vitremer ^b^	2. Nanohybrid composite: Tetric Ceram ^c^	Modified USPHS	1. Vitremer/35 2. Tetric Ceram/35	70/35	2 Years	Brazil/University	Split-mouth	93.3%	Visual-tactile using a mirror, and an explorer
Pollington et al.^48^	1. Compomer: Hytac ^b^	1. Universal Hybrid composite: Pertac II ^b^	Modified USPHS	1. Hytac/30 2. Pertac II/30	60/30	3 Years	United Kingdom/University	Split-mouth	100%	Visual-tactile (no details are mentioned)
Türkün et al.^46^	1. Compomer: Dyract ^e^	1. Nanofilled composite: Filtek Supreme ^b^	USPHS	1. Dyract/50 2. Filtek Supreme/50	100/24	2 Years	Turkey/University	Split-mouth	100%	Visual-tactile using a mirror, an explorer and radiographs
Gallo et al.^44^	1. Compomer: F 2000 ^b^	1. Microfilled composite: Silux Plus ^b^	Modified USPHS	1. F 2000 + Single bond (ER)/30 2. F 2000 + SE primer/30 3. Silux Plus + Single bond/30	90/30	3 Years	USA/University	Split-mouth	100%	Visual-tactile (No details are mentioned)
Onal et al.^45^	1. RMGIC: Vitremer ^b^ 2. Compomer: F 2000 ^b^ 3. Compomer: Dyract ^e^	1. Universal composite: Valus Plus ^b^	Modified USPHS	1. Vitremer /24 2. F 2000/38 3. Dyract 64 4. Valus Plus/22	130/30	2 Years	Turkey/University	Parallel ara>	93.8%	Visual-tactile (no details are mentioned)
Brackett et al.^42^	1. RMGIC: Fuji II LC ^a^	1. Microhybrid composite/Z250 ^b^	Modified USPHS	1. Fuji II LC/37 2. Z250/37	74/24	2 Years	Mexico/University	Split-mouth	73%	Visual-tactile (no details are mentioned)
Wucher et al.^47^	1. Compomer: Dyract ^e^	1. Microhybrid compiste: Spectrum TPH ^e^	USPHS	1. Dyract/23 2 Dyract covered with Spectrum /23 3. Spectrum TPH/23	69/23	3 Years	South Africa/Private practice	Split-mouth	86.9%	Visual-tactile using mirror, periodontal rob, and periapical radiographs at 1-year recalls
Folwaczny., et al.^54^	1. Compomer: Dyract ^e^ 2. RMGIC: Fuji II LC ^a^ 3. RMGIC: Photac Fil ^b^	1. Hybrid Compoiste: Tetric Ceram ^c^	Modified USPHS	1. Dyract/79 2. Fuji II LC/51 3. Tetric Ceram/36	197/37	2 Years	Germany/University setting	Parallel	N.r	Visual-tactile using mirrors and a prob

Study ID	10. Black’s classification	11. Cavity design and size	12. Gingival margin location/enamel bevel	13. Moisture control	14. Adhesive technique/Composite	15. Adhesive technique/Ion-releasing material	16. Patient’s age Mean ± SD [Range], in years			
Balkaya et al.^39^	Class II	Conservative slot design	Enamel/no bevel	Cotton pellets and suction	Single Bond Universal ^b^/SE	Polyacrylic acid conditioner ^a^	22^20–32^			
Gurgan et al.^19^	Class I and II	Conservative	Enamel/no bevel	Cotton rolls	G-bond ^a^/One step SE	Polyacrylic acid conditioner ^a^	24^15–37^			
Koc Vural et al.^37^	Class V (carious)	Conservative	Dentine/no bevel	Cotton rolls and saliva ejector	Prime & Bond NT ^e^/2-step ER	37% phosphoric acid for 5 s	52.69 ± 9.7^37–88^			
Koc Vural et al.^38^	Class V (NCCL)	Wedge shaped, and saucer-shaped	N.R	Cotton rolls and saliva ejector	Prime & Bond Elect One ^e^/Univeral adhseive with selective enamel etching	No preconditioning	55 ± 8.3^40,42–71^			
Miletić et al.^51^ara>	Class II	Conservative, moderate to large	Enamel/no bevel	Rubber dam for composite High suction and cotton roll for GIC	Adhese ^c^/2-step SE	Polyacrlic acid condition ^a^	> 18			
Oz et al.^53^	Class V (NCCL)	Non-retentive	Enamel + dentine/n.r	Cotton rolls	G-premio bond ^a^/Universal adhesive in ER mode	No pre-treatment	61.8 ± n.r Patient had at least one systemic disease			
Celik et al.^16^	Class V (NCCL)	Wedge or saucer-shaped	Dentine/no bevel	Cotton rolls, retraction cord, and a saliva aspirator	Optibond FL^d^/a 3-step ER	Polyacrylic acid ^a^	47.8 ± nr^34–62^			
Menezes-Silva et al.^49^	Class II	GIC/ATR Composite/conservative	Dentine/retention grooves for GIC	Cotton rolls	Single Bond Universal ^b^	Polyacrylic acid ^a^	N.r^8–19^			
Van Dijken et al.^41^	Class I and II	Retentive cavity	N.r/no bevel	Cotton rolls and suction	Xeno select ^e^/1- step SE	Etching for 5 s with phosphoric acid	58.3 ± n.r^37–85^			
Jassal et al.^50^	Class V (NCCL)	Non-retentive	Enamel + dentine/no bevel	Cotton rolls and retraction cord	G-bond ^a^/1- step SE	Polyacrylic acid	> 18			
Diem et al.^17^	Class I	Adhesive cavity preparation	No bevel	Cotton rolls	G-bond ^a^/1- step SE	Polyacrylic acid	N.r^11,12^ with occlusal caries in permanent first molars			
Van Dijken et al.^40^	Class V (carious)	Non-retentive	Dentine/no bevel	Cotton rolls and saliva suction device	Xeno III ^e^/1- step SE	Xeno III ^e^/1- step SE	61.5 ± n.r^40,43–83^			
Perdigão et al.^57^	Class V (NCCL)	Non-retentive	n.r/no bevel	Cotton rolls	FGM ^k^/2-step ER	1. Ketac Nano primer ^b^ . Polyacrylic acid conditioner with Fuji LC	48.7 ± n.r^30–78^			
De Moor et al.^43^	Class V (carious)	Conventional cavity preparation	Enamel/bevel	N.r	Optibond FL ^d^/3-step ER	Polyacrylic acid ^a^	45 [n.r] Head and neck cancer patients			
Santiago et al.^52^	Class V (NCCL)	Non-retentive	Enamel/no bevel	Rubber dam	Excite ^c^/2-step ER	Vitremer Primer ^b^	N.r ^18–50^			
Pollington et al.^48^	Class V (NCCL)	Non-retentive	Enamel + dentine/no bevel	Cotton rolls and high suction	Prompt L-Pop ^b^/1-step	Prompt L-Pop ^b^/1-step	54 [N.r]			
Türkün et al.^46^	Class V (NCCL)	Non-retentive	No bevel	Cotton rolls and retraction cord	Clearfil protect ^y^/2-step SE	Clearfil protect ^y^/2-step SE	44 ^25–54^			
Gallo et al.^44^	Class V (NCCL)	Non-retetnive	Enamel + dentine/bevel	Rubber dam	Single Bond ^b^/2-step ER	1. Single Bond ^b^/2-step ER 2. F 2000 ^b^/SE	N.r			
Onal et al.^45^	Class V (NCCL)	Non-retentive	Enamel + dentine/no bevel	Cotton rolls and suction	Scotchbond ^b^/3-step ER	1. Vitremer Primer ^b^ 2	N.r ^27–63^			
Brackett et al.^42^	Class V (NCCL)	Non-retentive	Enamel + dentine/n.r	Cotton rolls and retraction cord	Single Bond ^b^/3-step ER	Polyacrylic acid ^a^	47 ± n.r ^28–72^			
Wucher et al.^47^	Class II	Conventional design	N.r/no bevels	Cotton rolls and saliva ejector	Prime and Bond 2.1 ^e^/2-step ER	Prime and Bond 2.1 ^e^/2-step ER	N.r ^25–61^			
Folwaczny et al.^54^	Class V (carious and NCCL)	Non-retentive	Enamel + dentine/bevel	Cotton rolls	Syntac ^C^/3-step ER	PSA Dyract ^e^/2-step SE	N.r ^26–66^			

a: GC,Tokyo, Japan,

b: 3 M.

c: Heraeus Kulzer, Ha-nau, Germany.

d: Kerr—Sybron Gmbh, Karlsruhe, Germany.

e: Dentsply,Konstanz, Germany.

C: Vivadent, Schaan, Liechtenstein),

f: Pulpdent, Watertown, MA, USA).

j: SDI, Bayswater, Australia).

y: Kuraray; Osaka, Japan).

k: Joinville, Brazil.

n.r: not reported.

ER: Etch-and-rinse.

SE: Self-etch.

ART: atraumatic restorative technique.

RC: resin composite.

For resin composite,8 studies used ER adhesive system^16,37,42–45,47,52,57^ while 9 studies used SE adhesives^17,19,40,41,46,48,50,51,54^. Two studies used a universal adhesive in selective etch mode^38,49^, 1 in SE mode^39^, and 1 in ER mode^53^. For moisture control, cotton rolls and saliva ejectors were reported in the majority of studies except for 3 studies that used rubber dam isolation^44,51,52^.

Patients in all studies had no systemic diseases except two^43,53^. In one study^53^, patients were required to have at least one systemic disease and the other one^43^ included subjects who were xerostomic, head and neck, cancer patients who received radiation therapy. Ten studies^16,19,37–39,41,49–51,53^ were published in the years (2018–2020) with 6 in 2020, 3 in 2019, and 1 in 2018. No studies were identified from January to May of 2021. Five studies^17,40,43,52,57^ were published between 2010 and 2014. Seven studies^42,44–48,54^ were published before 2010.

### Descriptive analysis

**Figure 3:**
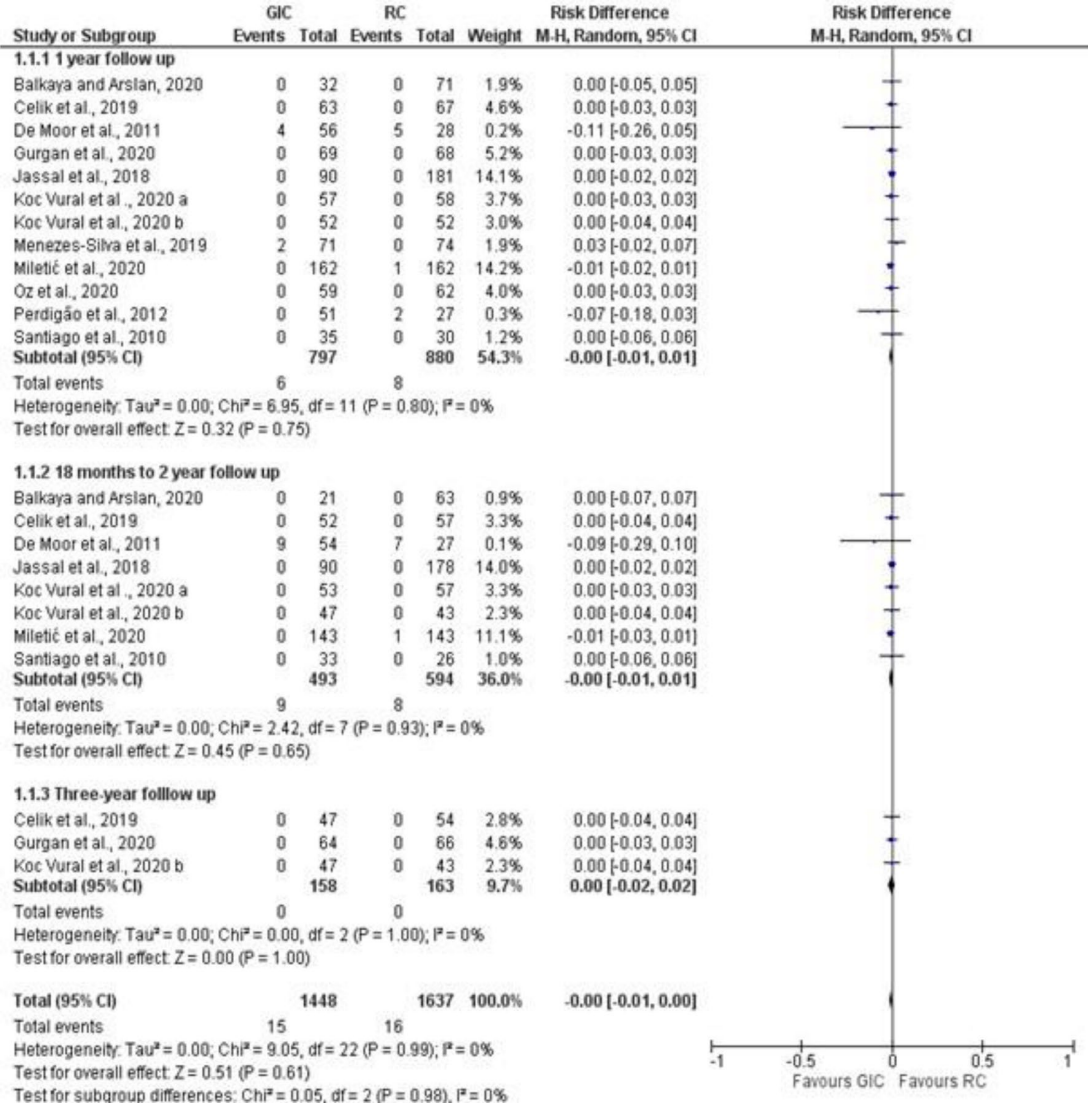
Forest plot of comparison: Ion releasing restoration (GIC) versus resin composite, outcome: 1.1 Secondary caries for all types of cavities.

**Figure 4:**
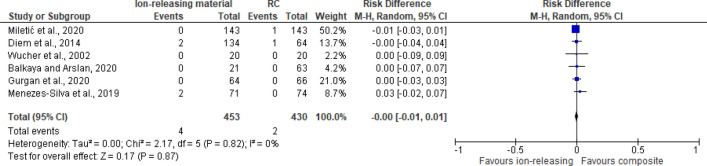
Forest plot of comparison: Ion releasing restoration versus resin composite, outcome: 1.2 Secondary caries for load-bearing cavities.

**Figure 5:**
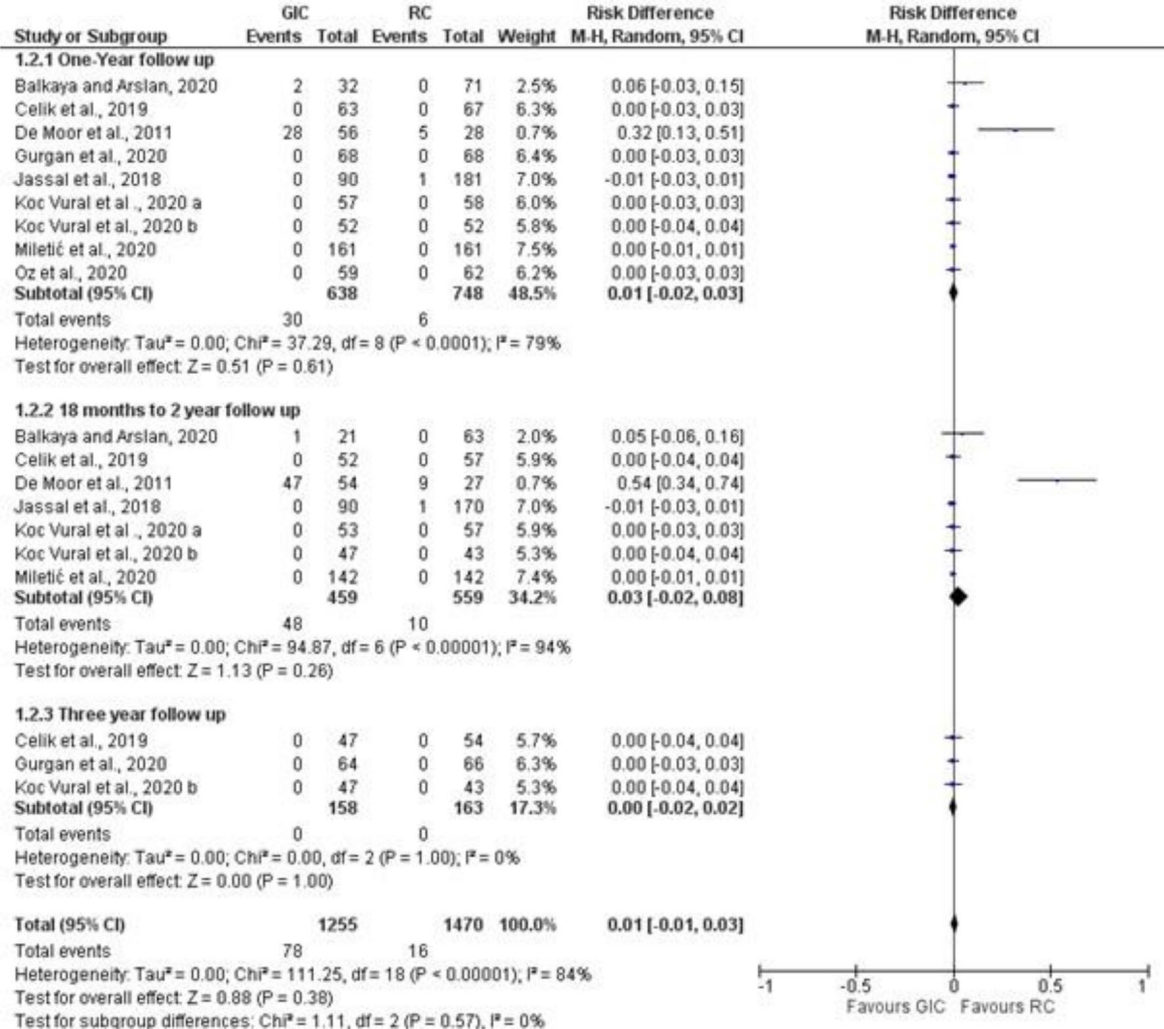
Forest plot of comparison: Ion releasing restoration (GIC) versus resin composite, outcome: 1.3 marginal adaptation.

Studies that reported secondary caries and marginal adaptation in different follow-up periods were included in the meta-analysis (Figs. 3, 4, 5). For secondary caries outcome for all types of cavities, the meta-analysis was grouped as follows: ion releasing materials (GIC) vs resin composite (RC) with the following 3 follow-up periods, i. e. 1 year, 18–24 months, and 3 years. For secondary caries in load-bearing cavities, ion-releasing material (GIC and compomer) vs resin composite, and data were extracted from the last follow-up.

The difference in the number of studies in each follow-up is attributed to whether the outcome was reported by the authors. For marginal adaptation outcome, GIC vs resin composite comparison was evaluated at the same 3 follow-up periods. secondary caries was not reported in all studies that compared compomer and resin composite at different follow-up periods. Therefore, no meta-analysis was performed for compomer vs resin composite comparison. Out of a total of 1448 GIC restorations, only 15 showed secondary caries with a percentage of 0.8%. Similarly, 16 composite restorations failed due to caries out of 1637 with a percentage of 0.9%. In all studies that compared compomer and resin composite, no occurrence of secondary caries was observed over the follow-up periods which ranged between 2 and 3 years.

### Meta-analysis

#### Secondary caries

The risk difference for the comparison between GIC and RC for the 1-year and18 month–2 years follow-up periods was -0.00 with 95% CI between [− 0.1–0.01]. The 3-year follow-up risk difference was 0.00 with 95% CI between [− 0.2–0.02] with no occurrence of secondary caries in both arms. There was no statistically significant difference (*P* = 0.61) between GIC and RC in secondary caries development at any of the follow-up periods. For a total of 1448 GIC restorations, 15 failed due to secondary caries, in comparison with 16 out of 1637 composite restorations. Overall heterogeneity was low with I^2^ = 0%. (Fig. 3). For secondary caries in load-bearing cavities, the risk difference was 0.0 with 95% CI between [− 0.01–0.02]. No statistically significant difference (*P* = 0.77) was found between ion-releasing material and secondary caries.

#### Marginal adaptation

The risk difference for the 1-year follow-up was 0.0.1 with 95% CI between [− 0.02–0.03]. Heterogeneity was high with an I^2^ = 75%. No statistically significant difference (*p* ˃ 0.5) was found between the 2 materials. At 18 months–2 years follow-up, the risk difference was 0.03 with 95% CI between [− 0.02–0.08]. Heterogeneity was high with an I^2^ = 94%. At the 3-year follow-up, the risk difference was 0.00 with 95%CI between [− 0.02–0.02]. Heterogeneity was low with an I^2^ = 0%.

The overall risk difference was 0.01 with 95%CI between [− 0.01–0.03]. Out of a total of 1255 GIC restorations, 78 showed unacceptable marginal adaptation compared to 16 out of 1470 RC restorations. No statistically significant difference was found between the 2 materials. Overall heterogeneity was high with an I^2^ = 84%. (Fig. 4).

#### GRADE quality of evidence

Assessment of the quality of evidence for secondary caries and marginal adaptation outcomes for the 3 follow-up periods (1 year, 18–24 months, and 3 years) was low. This finding suggests that the confidence in the effect estimate is limited, and that further research is likely to have an impact on the confidence of the estimate of effect (Table 2).

**Table 2:** Quality assessment of the included studies according to the GRADE tool.

Certainty assessment							Summary of findings				
Participants (studies) Follow up	Risk of bias	Inconsistency	Indirectness	Imprecision	Publication bias	Overall certainty of evidence	Study event rates (%)		Relative effect (95% CI)	Anticipated absolute effects	
							With resin composite restorations	With Ion releasing material (GIC)		Risk with resin composite restorations	Risk difference with Ion releasing material (GIC)
Secondary caries—1-year follow-up											
1677 (12 RCTs)	Serious^A^	Not serious	Not serious	Serious^B^	none	⨁⨁◯◯ LOW	8/880 (0.9%)	6/797 (0.8%)	Not estimable	9 per 1000	0 fewer per 1000 (from 10 fewer to 10 more)
Secondary caries—18 months to 2 years follow-up											
1087 (8 RCTs)	Serious^A^	Not serious	Not serious	Serious^B^	none	⨁⨁◯◯ LOW	8/594 (1.3%)	9/493 (1.8%)	Not estimable	13 per 1000	0 fewer per 1000 (from 10 fewer to 10 more)
Secondary caries—Three-year follow-up											
321 (3 RCTs)	Serious^A^	Not serious	Not serious	Serious^B^	none	⨁⨁◯◯ LOW	0/163 (0.0%)	0/158 (0.0%)	Not estimable	0 per 1000	0 fewer per 1000 (from 20 fewer to 20 more)
Marginal adaptation—One-Year follow-up											
1386 (9 RCTs)	Serious^A^	Not serious	Not serious	Serious^B^	none	⨁⨁◯◯ LOW	6/748 (0.8%)	30/638 (4.7%)	Not estimable	8 per 1,000	10 fewer per 1000 (from 30 fewer to 20 more)
Marginal adaptation—18 months to 2 years follow-up											
1018 (7 RCTs)	Serious^A^	Not serious	Not serious	Serious^B^	none	⨁⨁◯◯ LOW	10/559 (1.8%)	48/459 (10.5%)	Not estimable	18 per 1,000	30 fewer per 1000 (from 80 fewer to 20 more)
Marginal adaptation – Three-year follow-up											
321 (3 RCTs)	Serious^A^	Not serious	Not serious	Serious^B^	none	⨁⨁◯◯ LOW	0/163 (0.0%)	0/158 (0.0%)	Not estimable	0 per 1,000	0 fewer per 1000 (from 20 fewer to 20 more)

CI: Confidence interval.

A: most of the information is from studies with an unclear or high risk of bias.

B: Control and intervention arms had no events.

High quality: We are very confident that the true effect lies close to that of the estimate of the effect.

Moderate quality: We are moderately confident in the effect estimate: The true effect is likely to be close to the estimate of the effect, but there is a possibility that it is substantially different.

Low quality: Our confidence in the effect estimate is limited: The true effect may be substantially different from the estimate of the effect.

Very low quality: We have very little confidence in the effect estimate: The true effect is likely to be substantially different from the estimate of effect.

## Discussion

This systematic review discussed the occurrence of secondary caries in ion-releasing materials versus resin composite. Glass ionomer and its derivatives are the most clinically reported ion-releasing materials. Compomer was less frequently used. The results of the meta-analysis showed no significant difference between the secondary caries in resin composite and all derivatives of GIC.

Secondary caries is influenced by several factors with the most frequent ones being: the location of the lesion (cervical, proximal, or occlusal), patient’s caries risk, age, and socioeconomic status, operator’s skills variation, and detection methods and criteria^58^. The majority of studies included in this review were conducted in university settings with trained operators and under standardized conditions with patients who demonstrated moderate oral hygiene. This could explain the low number of events. Secondary caries was found to be more frequent in practice-based settings^7^. This could be attributed to the technique sensitivity of composite placement that requires highly skilled and calibrated operators which is often the case in university settings^59^. Regarding operative procedures, the majority of studies in this review used cotton rolls and saliva ejectors for moisture control while only 3 studies reported rubber dam isolation. Previous literature reported no significant difference between the survival of composite restorations performed under either of the isolation protocols^60^.

The location of the lesion is an important factor that could explain the generally low incidence of events. Around 45% of the included studies involved NCCL which are less affected by secondary caries than posterior occlusal and proximal cavities^59^. Secondary caries is reported to be more frequent with deep proximal restorations with gingival margins extending beyond the cementoenamel junction with dentine and cementum as the substrate^61,62^. Furthermore, the placement of such restorations is highly technique sensitive and isolation in every restorative step cannot be strictly followed^9^.

The Patient’s caries susceptibility is crucial in secondary caries development, as primary caries and secondary caries are inherently the same diseases and consequently patients with high caries risk are more suspectable to secondary caries^63^. The findings of this review were based on the results of studies performed on a population of healthy individuals with good to moderate oral hygiene and with no debilitating conditions. One exception is the study by De Moor et al.^43^, in which the population was head and neck xerostomic cancer patients who received radiation therapy. De Moor et al. ^43^, reported a significantly higher failure rate due to secondary caries in resin composite restorations in comparison with conventional GIC. Nevertheless, the findings of this study cannot be generalized as this population is highly specific. However, the difference in the performance of different materials in populations with compromised oral health indicates that patient factors could be more influential than the choice of material.

Adhesive strategy and interfacial gap formation were speculated to play a role in secondary caries development. Gaps at the margins of restorations can permit bacterial invasion and biofilm accumulation along the tooth/restoration interface^64^. However, until now there is no Conesus in the literature regarding the role of gaps in secondary caries development. In a study by Kidd et al.,^65^, it was suggested that microleakage cannot solely induce active demineralization beneath a restoration, only when bacterial invasion takes place at the composite-restoration interface, the size of the gap becomes pertinent.

The durability of the adhesive interface is critical for the survival of resin composite restorations, especially with dentin margins. Several attempts have been made to increase the durability of adhesives to dentine including using MMPs inhibitors, biomimetic remineralization, and increasing the hydrophobicity of the adhesive^66–68^. The adhesion protocols in this systematic review varied between etch-and-rinse (9 studies) and self-etch adhesives (8 studies), while 2 studies used universal adhesives in selective etch mode^38,49^, one in SE mode^39^ and one in ER mode^53^. The findings of this systematic review suggest that regarding secondary caries development, all adhesive strategies performed similarly considering the low number of events. In a previous study that utilized a short-term in vitro biofilm model^69^, the adhesive type affected carious lesion development and progression in gaps. However, a recent systematic review and Network meta-analysis showed similar performance of all adhesive strategies in preventing secondary caries^70^. It is worth mentioning that the impact of adhesive strategy/type on secondary caries development was not assessed quantitatively in this review, considering the overall scarcity of secondary caries occurrence in the included follow-up periods.

The short follow-up period (2–3 years) in the majority of studies might have contributed to an overall low incidence of events. Longer-term follow-up clinical trials showed an increased reporting of secondary carious lesions^71,72^. According to the findings of a recent review^59^, the highest mean incidence of secondary caries development was recorded after five years. Interestingly, the only long-term 10-year follow-up study for posterior restorations (class I and II) in this review^19^, did not report failure due to secondary caries for composites and glass ionomer restorations over the 10-year observational period. Furthermore, the detection methods and criteria of evaluation might have played a role in reporting secondary caries. According to a systematic review by Brouwer et al.^73^, only visual assessment would mean that 40% of secondary carious lesions will be missed, while 20% of sound surfaces will be misdiagnosed as carious. Until now, there is no clear consensus on what constitutes a secondary carious lesion that requires intervention^8,9^.

While the findings of in vitro studies^74,75^ reported a reduced risk of secondary caries in ion-releasing restorations such as GICs and their derivatives, the relation between the restorative material and secondary caries development is not clear in clinical settings. It is worth mentioning that clinical reporting in the form of randomized clinical trials on the recently developed ion-releasing materials is still scarce. Developments such as RMGIC with ionic resin matrix (Activa Bioactive) which is claimed to release ions in sufficient quantities to induce remineralization and inhibit secondary caries have not been thoroughly evaluated. The short-term performance was disappointing with an unacceptable failure rate due to the absence of an adhesive^41^. (a protocol no longer recommended by the manufacturer). Recent in vitro data regarding the ion-releasing Cention n showed its ability to neutralize the acidic environment^76^. However, no clinical evidence in the literature is available to validate the laboratory data.

The quality of the interface between the tooth structure and the restoration can play a significant role in the occurrence of secondary caries. While not the only route for secondary caries, the presence of a defective restoration margin can allow acidic fluids or biofilm to enter the interface via gaps. However, there is currently no agreement on the role of microleakage in the development of caries near composites. Nonetheless, some *in vivo* and *in vitro* studies suggest that the presence of a gap next to a composite restoration can result in the formation of a "wall lesion.". The literature also suggests the presence of a correlation between the size of the gap and the size of the dentinal wall lesions^59,64,77–79^.

The results of marginal adaptation between GIC derivatives showed comparable performance with resin composite restorations with no significant difference between them. Marginal adaptation of restorations is highly dependent on the quality of the adhesive interface^80,81^. Traditionally, attachment of resin composite restorations was achieved through micromechanical adhesion that involved the etching of the dental substrates^82^. Due to their user-friendly application, simplified universal adhesives have grown in popularity. According to the literature, these adhesives are a single-bottle, no-mix adhesive system that works well with any adhesion strategy and bonds adequately to tooth structure as well as various direct and indirect restorative materials^83,84^. However, the simplification came at the expense of hydrophilicity which can lead to water seepage through the hybrid layer causing nano leakage ^85^. Therefore, different protocols have been suggested to improve the performance of simplified adhesives including increasing the application time^86^, the addition of a hydrophobic resin layer over the adhesive^87^, and application of several layers of the simplified adhesive^88^. There is no clear consensus in the literature on the optimal way to improve the long-term performance of simplified adhesives.

The results of this systematic review showed a wide variation in the adhesion protocol for the ion-releasing materials, ranging from no pre-treatments to polyacrylic acid conditioners, ER, and SE adhesives. Nevertheless, the overall incidence of marginal deterioration was low. It is important to highlight that the adherent substrate which is a determining factor in the quality of the adhesion, is not consistent in all studies, with margins being in enamel, dentine, or cementum. GICs were applied in the majority of studies after pre-treatment with a cavity conditioner of poly-acrylic acid. It has been proposed that a tooth-GIC interaction interphase layer is seen after GIC comes in contact with pre-treated dentin, as the pre-treatment facilitates diffusion of ions into the demineralized substrate^89,90^.

A recent systematic review has shown that this interphase layer is notably resistant to acidic dissolution and hence improving the quality of the adhesive interface^91^. It is important to note that in this review, scores 1 and 2 of the FDI criteria in the marginal adaptation outcome were considered to be a sign of no significant marginal deterioration. This was done to distinguish early stages of marginal deterioration between ion-releasing materials and resin composite restorations. Since, the presence of small marginal gaps, ditches could potentially be a culprit in secondary caries development.

The risk of bias in more than 60% of the included studies was high, with only 3 studies reporting a low risk of bias^19,37,38^. Performance bias was high or unclear in most studies as the nature and presentation of the used materials are different and easily identified by dentists. It should be noted that the overall risk of bias of the study was not considered as a ground for meta-analysis exclusion. Therefore, the results of this analysis should be cautiously interpreted. The GRADE assessment of the quality of evidence was low for both outcomes (secondary caries and marginal adaptation) which weakens confidence in the effect estimate. Consequently, the true effect might be substantially different from the estimate of the effect. Imprecision and risk of bias for both outcomes had to be downgraded by one level each. The risk of bias for 2 of the primary domains (performance bias and selection bias) was high for studies that contributed to the weight of the analysis. The absence of events in control and intervention arms led to a downgrading for impression by one level^92^.

There are some limitations to this review. Firstly, no restriction was placed on the date of publication. Studies that were published in the early 2000s presented a higher risk of bias and inadequate reporting which affected their quality assessment. Although the Consolidated Standards of Reporting Trials (CONSORT) statement was developed in 1996^93^ and undergone a couple of revisions^94,95^, many clinical trial reports remained inadequate. Furthermore, short follow-up periods resulted in an overall low number of events. Also, several new ion-releasing materials have emerged in the last 5 years. The results of this analysis were based on two broad categories of materials (GICs and compomers). The findings of this review cannot be applied to all commercially available ion-releasing materials.

## Conclusions

1. Within the limitation of this work, this systematic review and meta-analysis revealed that secondary caries occurrence is not dependent on the ion-releasing capability of restorative material.
2. Short-term follow-ups are a common denominator among the available body of evidence. Longer follow-ups are recommended to accurately detect the performance of different restorative materials after prolonged clinical service.
3. Several new ion-releasing materials lack high-quality clinical reporting and need further investigations.

## Supplementary Information

Supplementary Information.

## Data Availability

The data used in this article are available upon request from the corresponding author.
