# Supplementary material for: Secondary caries and marginal adaptation of ion-releasing versus resin composite restorations: a systematic review and meta-analysis of randomized clinical trials
Source: Sci Rep. 2022 Nov 10;12:19244. doi: 10.1038/s41598-022-19622-6 (PMC9649593; doi:10.1038/s41598-022-19622-6)
Supplement: Supplementary file 1 — Supplementary Information. [file 41598_2022_19622_MOESM1_ESM.doc]

**Secondary caries and marginal adaptation of ion-releasing vs resin composite restorations: a systematic review and meta-analysis of randomized clinical trials**

**Eman H. Albelasy ^1,2^,** **Hamdi H. Hamama^1^, Hooi Pin Chew^3^, Marmar Montaser ^1^, Salah H. Mahmoud ^1^**

^1^. Operative Dentistry Department, Faculty of Dentistry, Algomhoria Street, Mansoura, Aldakhlia, Egypt Po (box) 35516

^2^ Research visiting scholar, Minnesota Dental Research Centre for Biomaterials and Biomechanics, School of Dentistry, University of Minnesota, Minneapolis, MN55455, USA

^3^ Minnesota Dental Research Centre for Biomaterials and Biomechanics, School of Dentistry, University of Minnesota, Minneapolis, MN55455, USA

*** Corresponding Author**

Hamdi H Hamama

Operative Dentistry Department, Faculty of Dentistry, Mansoura University, Egypt.

Mailing address: Algomhoria St, Mansoura, Aldakhlia, Egypt
Postal Code (35516)

Tel: +002 (01153418154)

Email: **hamdy@connect.hku.hk**

**Table S1: PRISMA 2009 checklist**

| **Section/topic** | **#** | **Checklist item** | **Reported on page #** |
| --- | --- | --- | --- |
| **TITLE** | | |  |
| Title | 1 | Identify the report as a systematic review, meta-analysis, or both. | 1 |
| **ABSTRACT** | | |  |
| Structured summary | 2 | Provide a structured summary including, as applicable: background; objectives; data sources; study eligibility criteria, participants, and interventions; study appraisal and synthesis methods; results; limitations; conclusions and implications of key findings; systematic review registration number. | 5 |
| **INTRODUCTION** | | |  |
| Rationale | 3 | Describe the rationale for the review in the context of what is already known. | 7 |
| Objectives | 4 | Provide an explicit statement of questions being addressed with reference to participants, interventions, comparisons, outcomes, and study design (PICOS). | 7 |
| **METHODS** | | |  |
| Protocol and registration | 5 | Indicate if a review protocol exists, if and where it can be accessed (e.g., Web address), and, if available, provide registration information including registration number. | PROSPERO, 8 |
| Eligibility criteria | 6 | Specify study characteristics (e.g., PICOS, length of follow-up) and report characteristics (e.g., years considered, language, publication status) used as criteria for eligibility, giving rationale. | 8 |
| Information sources | 7 | Describe all information sources (e.g., databases with dates of coverage, contact with study authors to identify additional studies) in the search and date last searched. | 8 |
| Search | 8 | Present full electronic search strategy for at least one database, including any limits used, such that it could be repeated. | Table S1 |
| Study selection | 9 | State the process for selecting studies (i.e., screening, eligibility, included in systematic review, and, if applicable, included in the meta-analysis). | 8 |
| Data collection process | 10 | Describe method of data extraction from reports (e.g., piloted forms, independently, in duplicate) and any processes for obtaining and confirming data from investigators. | 8 |
| Data items | 11 | List and define all variables for which data were sought (e.g., PICOS, funding sources) and any assumptions and simplifications made. | 8 |
| Risk of bias in individual studies | 12 | Describe methods used for assessing risk of bias of individual studies (including specification of whether this was done at the study or outcome level), and how this information is to be used in any data synthesis. | 8 |
| Summary measures | 13 | State the principal summary measures (e.g., risk ratio, difference in means). | 8 |
| Synthesis of results | 14 | Describe the methods of handling data and combining results of studies, if done, including measures of consistency (e.g., I^2^) for each meta-analysis. | 8 |

Page 1 of 2

| **Section/topic** | **#** | **Checklist item** | **Reported on page #** |
| --- | --- | --- | --- |
| Risk of bias across studies | 15 | Specify any assessment of risk of bias that may affect the cumulative evidence (e.g., publication bias, selective reporting within studies). | - |
| Additional analyses | 16 | Describe methods of additional analyses (e.g., sensitivity or subgroup analyses, meta-regression), if done, indicating which were pre-specified. | - |
| **RESULTS** | | |  |
| Study selection | 17 | Give numbers of studies screened, assessed for eligibility, and included in the review, with reasons for exclusions at each stage, ideally with a flow diagram. | Figure 1 |
| Study characteristics | 18 | For each study, present characteristics for which data were extracted (e.g., study size, PICOS, follow-up period) and provide the citations. | Table 1 |
| Risk of bias within studies | 19 | Present data on risk of bias of each study and, if available, any outcome level assessment (see item 12). | Table S4 |
| Results of individual studies | 20 | For all outcomes considered (benefits or harms), present, for each study: (a) simple summary data for each intervention group (b) effect estimates and confidence intervals, ideally with a forest plot. | Figure 2 |
| Synthesis of results | 21 | Present results of each meta-analysis done, including confidence intervals and measures of consistency. | Figure 2 |
| Risk of bias across studies | 22 | Present results of any assessment of risk of bias across studies (see Item 15). | - |
| Additional analysis | 23 | Give results of additional analyses, if done (e.g., sensitivity or subgroup analyses, meta-regression [see Item 16]). | - |
| **DISCUSSION** | | |  |
| Summary of evidence | 24 | Summarize the main findings including the strength of evidence for each main outcome; consider their relevance to key groups (e.g., healthcare providers, users, and policy makers). | 10 |
| Limitations | 25 | Discuss limitations at study and outcome level (e.g., risk of bias), and at review-level (e.g., incomplete retrieval of identified research, reporting bias). | 10-11 |
| Conclusions | 26 | Provide a general interpretation of the results in the context of other evidence, and implications for future research. | 11-12 |
| **FUNDING** | | |  |
| Funding | 27 | Describe sources of funding for the systematic review and other support (e.g., supply of data); role of funders for the systematic review. | 14 |

**Table S2. Excluded studies at the full-text assessment stage.**

| Study ID | Title | Reasons for exclusion |
| --- | --- | --- |
| Di Lenarda et al., [1] | Cervical compomer restorations: the role of cavity etching in a 48-month clinical evaluation | No resin composite restorations were used as a control |
| Ermiş et al., [2] | Two-year clinical evaluation of four polyacid-modified resin composites and a resin-modified glass-ionomer cement in Class V lesions. | No resin composite restorations were used as a control |
| Favetti et al., [3] | Effects of cervical restorations on the periodontal tissues: 5-year follow-up results of a randomized clinical trial | No ion-releasing material was used. |
| Lund et al., [4] | Clinical Performance and Wear Resistance of Two Compomers in Posterior Occlusal Restorations of Permanent Teeth: Six-Year Follow-up | NO resin composite restorations were used |
| Fotiadou et al., [5] | A 3-year controlled randomized clinical study on the performance of two glass-ionomer cements in Class II cavities of permanent teeth | No resin composite restorations were used |
| Lindberg et al., [6] | Nine-year evaluation of a polyacid-modified resin composite/resin composite open sandwich technique in Class II cavities | Polyacid-modified resin composite was placed under resin composite, so secondary caries associated with it can not be assessed. |
| Klinke et al., [7] | Clinical performance during 48 months of two current glass ionomer restorative systems with coatings: a randomized clinical trial in the field | No resin composite was used as a control |
| Konde et al., [8] | Clinical evaluation of a new art material: Nanoparticulated resin-modified glass ionomer cement | 1.The study was conducted on primary molars.  2. No resin composite was used as a control |
| Loguercio et al., [9] | Five-year double-blind randomized clinical evaluation of a resin-modified glass ionomer and a polyacid-modified resin in noncarious cervical lesions | No resin composite was used as a control |
| Mandari et al., [10] | Effectiveness of three minimal intervention approaches for managing dental caries: survival of restorations after 2 years | Amalgam was used as a control |
| Mobarak et al., [11] | Survival of occlusal ART restorations using high-viscosity glass-ionomer with and without chlorhexidine: A 2-year split-mouth quadruple-blind randomized controlled clinical trial | No resin composite was uses as a control |
| Oberländer et al., [12] | Clinical performance of polyacid-modified resin restorations using "soft start-polymerization" | No resin composite was used as a control |
| Priyadarshini et al., [13] | One-year comparative evaluation of Ketac Nano with resin-modified glass ionomer cement and Giomer in non- cervical lesions: A randomized clinical trial | No resin composite was used as a control |
| Oz et al., [14] | Clinical evaluation of a self-adhering flowable resin composite in minimally invasive class i cavities: 5-year results of a double blind randomized, controlled clinical trial | No ion-releasing restorative material was used for comparison |
| Crisp et al., [15] | One-year clinical evaluation of compomer restorations placed in general practice | No resin composite restorations were used |
| Brackett et al., [16] | Two-year clinical performance of a resin-modified glass-ionomer restorative material | No resin composite restorations were used |
| Burke et al., [17] | Clinical performance of reinforced glass ionomer restorations placed in UK dental practices | No resin composite was used |
